# Analysis of *WAK* Genes in Nine Cruciferous Species with a Focus on *Brassica napus* L.

**DOI:** 10.3390/ijms241713601

**Published:** 2023-09-02

**Authors:** Zishu Xu, Yi Duan, Hui Liu, Mingchao Xu, Zhi Zhang, Ling Xu

**Affiliations:** 1 Key Laboratory of Plant Secondary Metabolism and Regulation of Zhejiang Province, College of Life Sciences and Medicine, Zhejiang Sci-Tech University, Hangzhou 310018, China; 2 UWA School of Agriculture and Environment and The UWA Institute of Agriculture, Faculty of Science, The University of Western Australia, Perth, WA 6009, Australia; 3 Leshan Academy of Agricultural Sciences, Leshan 614000, China

**Keywords:** *Brasscia napus* L., wall-associated kinase, cruciferae, expression profile, gene family, bioinformatics analysis

## Abstract

The wall-associated kinase family contributes to plant cell elongation and pathogen recognition. Nine Cruciferous species were studied for identification and molecular evolution of the *WAK* gene family. Firstly, 178 *WAK* genes were identified. A phylogenetic tree was constructed of the Cruciferous *WAK* proteins into four categories, of which the *Brassica rapa*, *Brassica oleracea* and *Brassica napus* genes in the U’s triangle were more closely related. The *WAK* gene family was unevenly distributed in *B. napus* chromosomal imaging, with the largest number of *BnWAK* genes located on chromosome C08. In the expression analysis, the expression patterns of the *WAK* gene family varied under different stress treatments, and some members of *BnWAK*s were significantly different under stress treatments. This study lays a foundation for further revealing the functional mechanisms of the *WAK* gene family in *Brassica napus*.

## 1. Introduction

During the latter part of the 1990s, the wall-associated kinase (*WAK*) was identified in Arabidopsis as a constituent of a diminutive gene cluster situated on chromosome 1 [1]. In plants, *WAK* is one of the genes associated with the cell wall, which plays an essential role in monitoring and interacting with the extracellular environment [2,3]. The *WAK* gene family belongs to distinct subgroups within the receptor-like protein kinase (RLK) superfamily. They share a well-conserved Ser/Thr domain and numerous epidermal growth factor (EGF) repeats [4,5,6,7]. *WAK*s bind and respond to pectin with their unique EGF repeats, and evidence supports the conjecture that the interaction with negatively charged pectin is dependent upon the positively charged conserved lysine residues of EGF repeats [1,3,5,8]. However, it is still questionable how *WAK*s bind to pectin, and this interaction may be related to sugar modification, which carries predicted N- and O-linked glycosylation signals [1].

Found in both dicots and monocots, there have been more and more reports of related *WAK* genes in plants [2,5]. In addition to their role in cell expansion, *WAK*s play an essential role in cell communication, as well as contributing to plant immunity in various ways [7,9]. *WAK*s have only recently gained prominence in plant immunity as vital players [10]. In some cases, these receptors are localized on the cell surface and recognize invasion molecules including effectors or damage-associated molecular patterns, whereas in others, the cell wall is modified and strengthened, thereby increasing the synthesis of cellulose and a plant antitoxin and oxidative burst, thereby limiting the intrusion of pathogens [3,11,12]. Various cereal crops have been reported to be tolerant to fungal and bacterial diseases through *WAK*s, including *ZmWAK* (*qHSR1*) [13], *ZmWAK-RLK1* (*Htn1*) [14] and *OsWAK* (*Xa4*) [15]. These genes are resistant to maize head smut, northern maize leaf blight (NCLB) and rice bacterial blight. Moreover, the *WAKs* of dicotyledons play an essential role in immunity as well. Through its complexation with the chitin receptors, a pivotal role is played by *GhWAK7A* in the response of cotton to fungal wilt pathogens [16]. For cotton to be more resistant to Verticillium wilt, *GhWAKL* is crucial [17].

A few researchers have studied the molecular functions of *WAK* genes in recent years, but a report on their molecular evolution is unavailable. There are many different types of crops in the world, such as rice, wheat, corn, rapeseed, cabbage and soybeans [18,19,20,21,22,23,24,25]. And the Brassicaceae family includes various economic plants for edible or ornamental purposes [26]. As currently delimited, the Brassicaceae includes 349 genera and 4060 species [27]. Brassicaceae vegetables are considered to be a significant part of the human diet due to their phytochemical content [26]. As a crucial member of Brassicaceae, *Brassica napus.* L. is a crucial oil crop, considered the third vegetable oil resource only after soybeans and palm oil, and produces 13% of total edible oil around the world [28,29,30,31]. However, its productivity has been reduced with many environmental adversities [32], such as salt [26], pathogens [33,34] and heat [35]. A crucial component of plant immunity, *WAK*s are essential to the quality and yield of *B. napus*. The study of the *WAK* gene family will provide insight into genetic breeding of oilseed resistance as well as provide a basis for a functional analysis of *WAK* genes.

## 2. Results

### 2.1. Analysis of the WAK Protein Phylogeny and Characterization

It has been found that *B. napus* has a total of 36 *WAK* genes, and each protein contains at least one *WAK* domain (Table S1). Among the *BnWAK* genes, the length ranges from 3022 bp (*BnWAK5*) to 661 bp (*BnWAK21*), and the length of the corresponding proteins ranges from 990 (*BnWAK5*) to 216 (*BnWAK21*) aa residues. Among the *WAK* proteins, the prediction of molecular weight varies between 109.7 kDa (*BnWAK5*) and 23.7 kDa (*BnWAK21*), while the theoretical pI varies between 4.96 (*BnWAK18*) and 7.91 (*BnWAK5*) (Table S1). According to the predicted subcellular localization, all *BnWAK* proteins are located extracellularly, consistent with the physiological function of cell receptors. The *WAK* gene family was further analyzed in eight other species in the Cruciferae family and 16 were found in *Arabidopsis helleri*, 16 in *Arabidopsis lyrata*, 16 in *Arabidopsis thaliana*, 7 in *Arabis alpina*, 19 in *Brassica juncea*, 26 in *Brassica oleracea*, 13 in *Brassica rapa* and 33 in *Camelina sativa* (Table S1).

**Figure 1:**
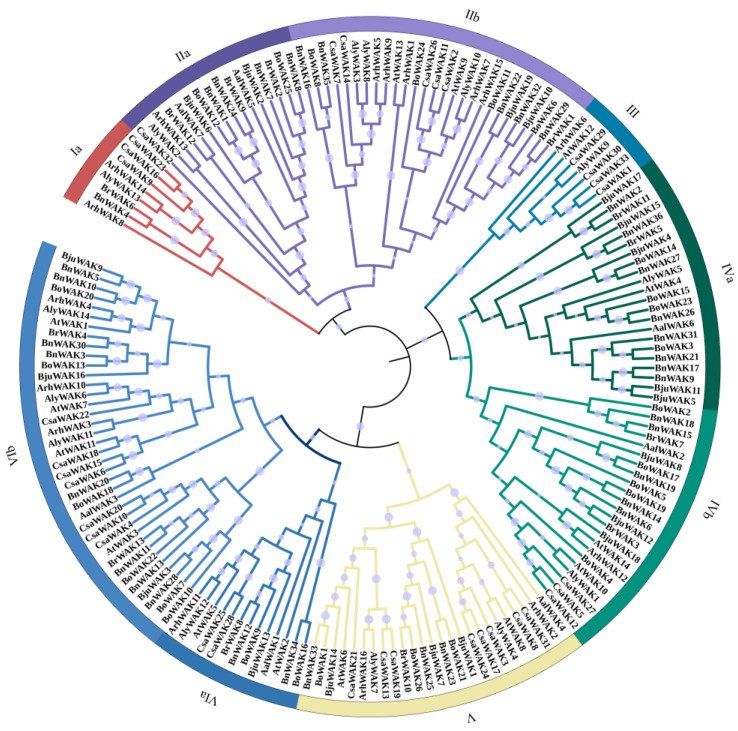
Phylogenetic analysis of 9 Cruciferous species’ *WAK* members. According to the alignment of the *WAK* domain, a phylogenetic tree was constructed using the neighbor-joining (NJ) method with the default parameters and 1000 bootstrap replicates. Based on the results of phylogenetic analysis, the *WAK* members of 9 cruciferous species were divided into 9 subfamilies, denoted by the Roman numerals from Ia to IVb.

The evolutionary relationship between *WAK* genes in *B. napus* and other Cruciferous plants was investigated by constructing a rooted phylogenetic tree of *WAK*s, including *B. napus* (36), *A. helleri* (16), *A. lyrata* (16), *A. thaliana* (16), *A. alpina* (7), *B. juncea* (19), *B. oleracea* (26), *B. rapa* (13) and *C. sativa* (33). Based on a phylogenetic analysis, *WAK* proteins clustered spontaneously into nine clades, and *WAK* was unevenly distributed in these clades (Figure 1). A total of 8 to 50 members were found within these clades, with clade VB containing the highest number of *WAK* genes. Among these plant species, clade I contained the lowest number of *WAK* family genes. *B. napus*, *B. juncea*, *B. oleracea* and *B. rapa* are typically clustered together, even within the same subfamily. In the IVb subfamily, for example, all these plants were grouped together. 

### 2.2. Gene Organization and Conserved Motif of the WAK Gene Family in B. napus

**Figure 2:**
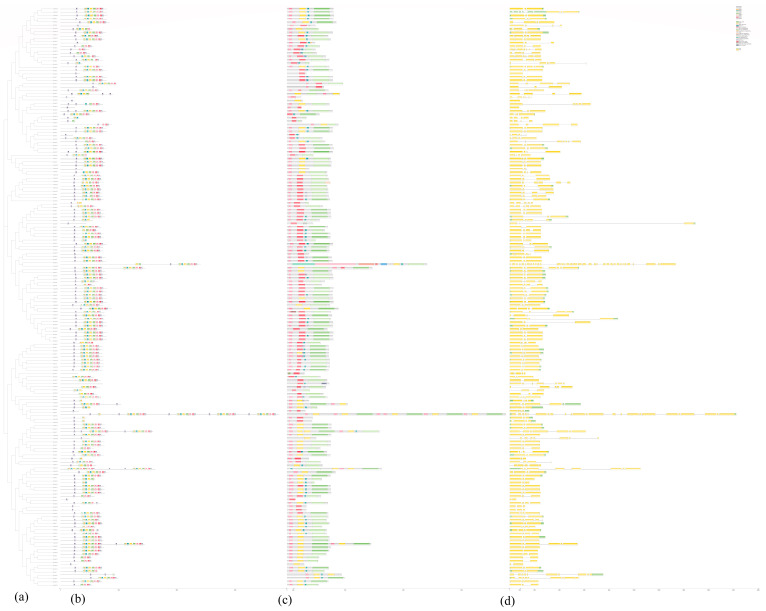
An ML phylogenetic tree with conserved motifs, gene structures and conserved domains of *WAK* proteins. (**a**). ML phylogenetic tree based on the *WAK* proteins’ domain sequence. (**b**). Ten conserved motifs of 178 *WAK* proteins from 9 Cruciferous species. The MEME program identified 10 conserved motifs, each characterized by a distinct color. Motif 9 constitutes key components of the *WAK* structural domain. (**c**). Conversed structural domains of 178 *WAK* genes for 9 Cruciferous species. *WAK* conserved structural domains are mapped directly to the gene structure. (**d**). Exon/intron structure of 178 *WAK* genes for 9 Cruciferous species. The green region represents the UTR, the yellow region represents the CDS and the gray lines indicate the exon regions, where exons partition most of the *WAK* conserved structural domains.

The structure and motif distribution of *WAK* genes were analyzed to investigate their characteristics (Figure 2). Utilizing the protein sequences of nine Cruciferous species, conserved motifs were identified through the MEME program, and 10 conserved motifs were identified in total (Figure 2b). A MEME analysis showed that *WAK* genes had minor differences in motif composition and quantity, with conserved motifs ranging from 1 to 43. There is a large presence of motif 9 and motif 7 among all *WAK* proteins, which were annotated as core motifs (Figure 2b). While motif distributions vary, they tend to be highly consistent within the same group (Figure 2b). As an example, motif 9 was primarily present among members of a clade within subfamily Vb, whereas motif 9 was present among all members of group Ia. Group Ⅲ comprised ten motifs, while subgroup Ⅳb contained conserved motifs 7, 8 and 9.

Additionally, it was revealed that *WAK* genes were composed of more exons based on the predicted gene structure (Figure 2d). GFF annotation results indicated that the relative number of exons varied from 3 to 36 in these *WAK*s. In addition, 56 *WAK*s contain introns. These 56 genes are widely present in all groups. The majority of family members contain between 3 and 10 exons, while *BoWAK*24 consists of 36 exons and *CsaWAK*3 consists of 25 exons. Most members of the same subfamily tend to be more similar in terms of the structure of their exons and introns as well as their intron phase distribution. As a result, the validity of classifications based on the phylogenetic analysis can be further supported.

### 2.3. Chromosomal Localization and Collinearity Analysis of BnWAKs

**Figure 3:**
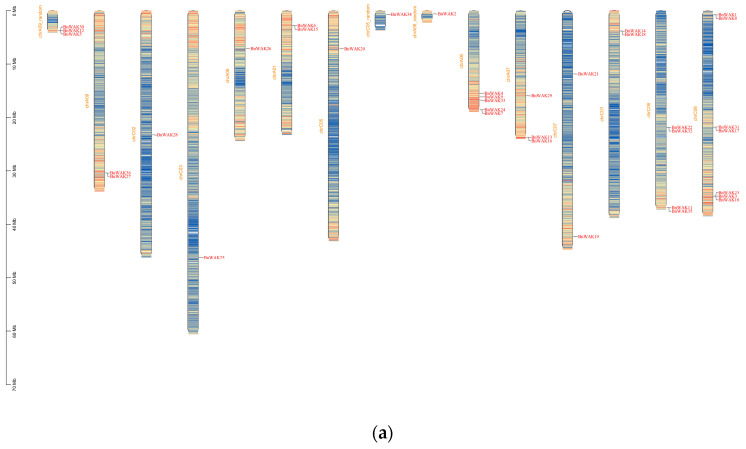

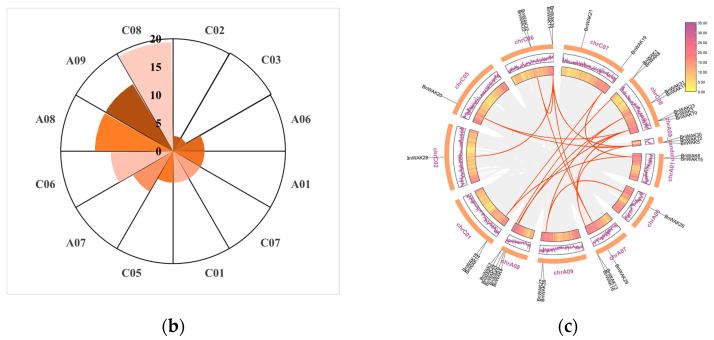
Chromosomal distribution and gene duplication events of *BnWAK*s. (**a**). *BnWAK*s’ position on the chromosome. The chromosome, in this instance, is divided into units of 0.1MB each. The density of the region is represented by the color variation, where blue indicates low, yellow indicates medium and red indicates high density. The physical location of the *BnWAK*s is represented by blue lines on the chromosome. (**b**). The quantity of *BnWAK*s on chromosomes. Each chromosome is represented by different colors. There are three genes that are not located on the chromosome. Therefore, large wedges imply a greater number of *BnWAK* genes. (**c**). A genome-wide analysis of gene synteny in *B. napus*. Synteny relationships are depicted by gray lines for each gene in *B. napus*, while replication events are represented by red lines.

According to information about physical location, the *BnWAK* genes were located on the 12 chromosomes of *B. napus* (Figure 3). *BnWAK* genes are evenly distributed across chromosomes. There were 17 *BnWAK*s on the A subgenome and 19 *BnWAK*s on the C subgenome. It was found that there were 1 to 7 *BnWAK* genes on each chromosome, with the densest distributions occurring on ChrC08 (7), then ChrA08 (5) and ChrC06 (4). There are also some regions, including ChrC02, ChrC03 and ChrC05, with only one *BnWAK* gene. Out of 36 *BnWAK* genes, the exact chromosomal positions of the 5 genes located on A06_random, A09_random and C05_random were unknown. In *B. napus*, the quantity of *WAK* genes is not significantly correlated with chromosome length.

The MCScanx gene duplication analysis was performed to explore *WAK* genes’ expansion in *B. napus*. The gene duplication analysis detected 18 pairs of gene duplication events in total (Figure 3c). Among these pairs, one was a tandem duplication (*BnWAK22/BnWAK35*), located on ChrC06, while the remainders were segmental duplication genes, mostly located on ChrC08 (5/7), ChrA07 (3/3), ChrA08 (3/5) and ChrA09 (2/2). The evolution of *BnWAK* genes, particularly those located on ChrC08, ChrA07, ChrA08 and ChrA09, was significantly influenced by segmental duplication. *Ka/Ks* was applied to 18 gene pairs in order to analyze the forces that drove gene evolution. The ratios of *Ka/Ks* for the 18 replicated gene pairs indicated that *BnWAK* development was significantly impacted by purified selection.

### 2.4. Cis-Acting Element Analysis of WAK Promoters in B. napus

**Figure 4:**
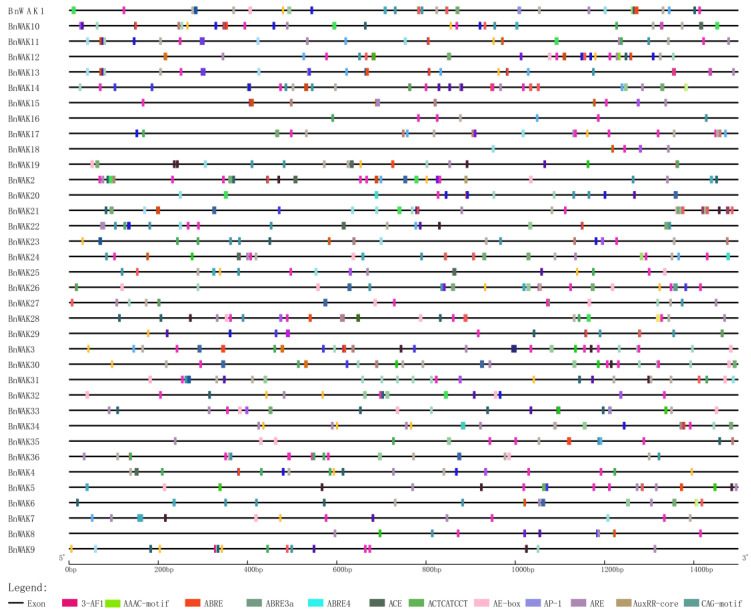
Cis-regulatory elements in the promoter region of the *BnWAK* genes. The prediction of cis-regulatory elements for 1.5 kb sequences upstream of *BnWAK*s. And some of the stress response elements and hormone response elements are finally shown.

To analyze the potential regulation and functions of *WAK* genes, cis-acting elements (CREs) were analyzed on promoter sequences (Figure 4). In the promoter region, abundant CREs were found, such as stress-responsive elements and hormone-responsive elements. Most of these elements were light-responsive elements, suggesting that light signals are significant regulators of plant growth. Additionally, some elements associated with endogenous hormones including MeJA, gibberellin, auxin, abscisic acid, low temperature, drought, defense and stress, and other environment-related elements, and meristem response elements were also presented. Moreover, *MYB*, the largest transcription factor family in plants, has also been observed to possess CREs in large quantities as a result of various stresses encountered during the plant development. 

In total, 853 CREs, which were related to response and hormonal stress, were selected to predict the underlying mechanisms of *BnWAK* genes under abiotic and hormonal treatment (Figure 4). A total of 132 methyl jasmonate (MeJA) CREs were found among several hormone-associated CREs, suggesting that this hormone is critical to plant growth. Additionally, the remaining hormone CREs were abscisic acid (ABA, 61), indole-3-acetic acid (IAA, 16), gibberellin (GA, 24) and salicylic acid (SA, 19). Multiple hormone response elements in gene promoter regions are generally associated with heightened hormone sensitivity. The balance of hormones may be regulated with this element, e.g., *BnWAK3* contains 12 MeJA elements, *BnWAK26* includes 10 MeJA elements and *BnWAK30* contains 10 MeJA elements. In addition, a selection of CREs associated with the growth and development of plants, such as those pertaining to cell division organization and circadian rhythms, were also chosen. This implies that the genes in which these cis-regulatory elements are located tend to have specific functions and that these genes are relatively conserved in plants.

### 2.5. Interspecies Syntenic Analysis in Cruciferous Species

**Figure 5:**
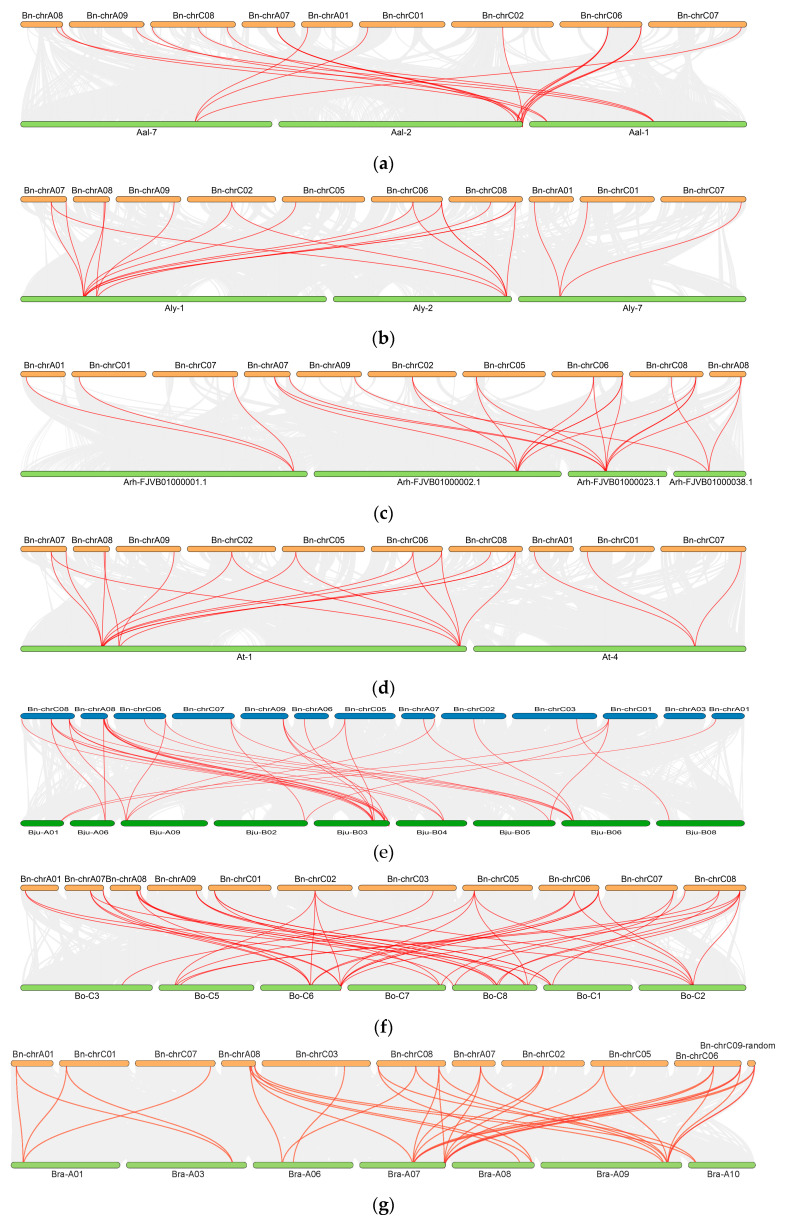

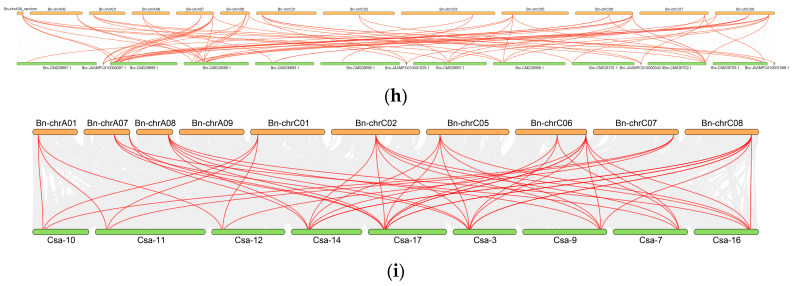
Synteny analyses of 8 dicotyledonous for *WAK* genes. The syntenic analysis between *B. napus* and the other eight dicotyledonous including *A. thaliana*, *A. lyrata*, *A. halleri*, *A. alpina*, *C. sativa*, *B. carinata*, *B. juncea*, *B. rapa* and *B. oleracea*. Collinear blocks are presented with gray lines shown in the background, while red lines highlight *WAK* gene pairs with syntenic relationships. (**a**) The syntenic analysis between *B. napus* and *A. alpina* (**b**) The syntenic analysis between *B. napus* and *A. lyrata* (**c**) The syntenic analysis between *B. napus* and *A. halleri* (**d**) The syntenic analysis between *B. napus* and *A. thaliana* (**e**) The syntenic analysis between *B. napus* and *B. juncea* (**f**) The syntenic analysis between *B. napus* and *B. oleracea* (**g**) The syntenic analysis between *B. napus* and *B. rapa* (**h**) The syntenic analysis between *B. napus* and *B. carinata* (**i**) The syntenic analysis between *B. napus* and *C. sativa*.

To investigate the homology and evolutionary relationships between *WAK* genes, nine Cruciferous species (*A. thaliana*, *A. lyrate*, *A. helleri*, *A. alpina*, *C. sativa*, *B. juncea*, *B. rapa*, *B. carinata* and *B. oleracea*) and *B. napus* were subjected to a synteny analysis (Figure 5). The results showed that *BnWAK* genes were homologous in all nine species, with 20 pairs with *A. alpina* (Figure 5a), 22 pairs with *A. lyrate* (Figure 5b), 25 pairs with *A. helleri* (Figure 5c), 23 pairs with *A. thaliana* (Figure 5d), 41 pairs with *B. juncea* (Figure 5e), 50 pairs with *B. oleracea* (Figure 5f), 36 pairs with *B. rapa* (Figure 5g), 65 pairs with *B. carinata* (Figure 5h) and 56 pairs with *C. sativa* (Figure 5i), suggesting that these *WAK* gene pairs may have come from a common ancestor and had the same function.

In the case of the crosstalk between *B. napus* and *B. carinata*, individual homologous genes displayed a purity of one-to-many or many-to-one. Several *WAK* genes seem to have contributed to the evolution of plants with U’s triangular. Several proteins of unknown function in *B. napus* can be predicted based on these results.

### 2.6. Orthologous Gene Clustering of WAK Gene Family in Five Cruciferous Species

**Figure 6:**
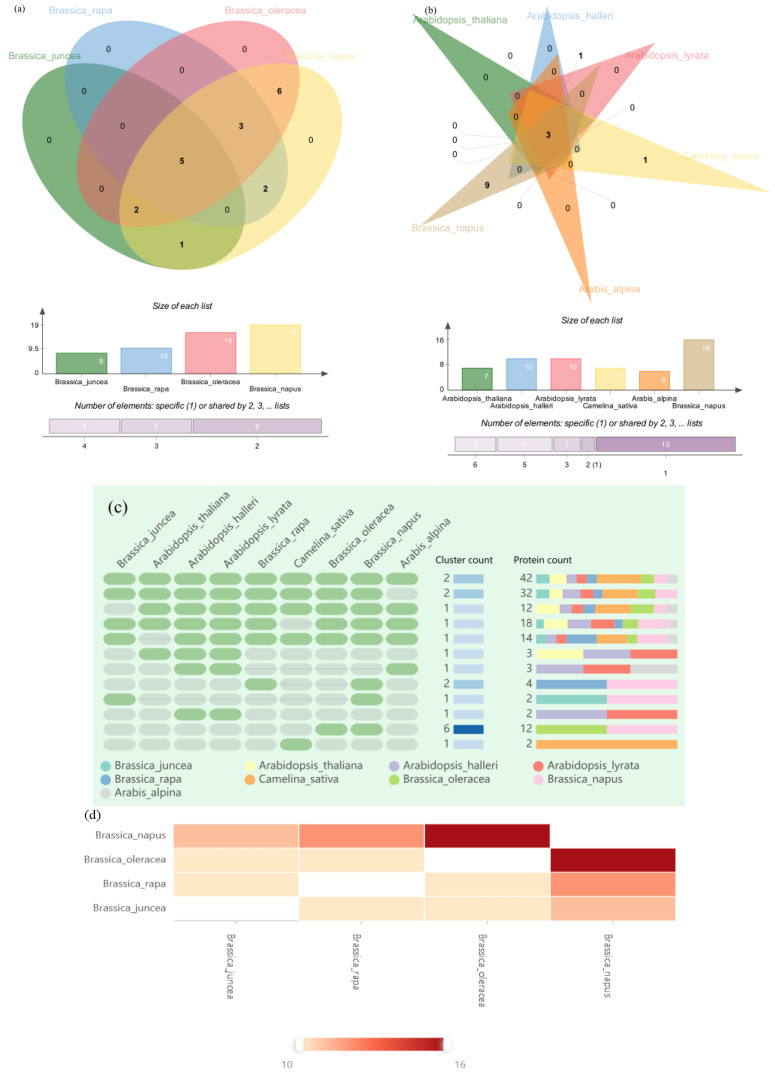
Orthologous gene clustering analysis. The orthologous gene clusters between the *WAK* gene family in *A. thaliana*, *A. lyrata*, *A. halleri*, *A. alpina*, *C. sativa*, *B. napus*, *B. juncea*, *B. rapa* and *B. oleracea* were identified and visualized using the OrthoVenn2 web platform. (**a**) Orthologous gene clustering analysis among the *WAK* gene family in *B. napus*, *B. juncea*, *B. rapa* and *B. oleracea* (**b**) Orthologous gene clustering analysis among the *WAK* gene family in *B. napus*, *A. thaliana*, *A. lyrata*, *A. halleri*, *A. alpina*, and *C. sativa.* (**c**) Orthologous gene clustering analysis among the *WAK* gene family in 9 Cruciferous species (**d**) Pairwise heatmap among the *WAK* gene family in *B. napus*, *B. juncea*, *B. rapa* and *B. oleracea*.

To explore the evolutionary relationships of *WAK* genes among nine species of Cruciferous plants—*A. thaliana*, *A. lyrate*, *A. helleri*, *A. alpina*, *C. sativa*, *B. juncea*, *B. rapa*, *B. carinata*, *B. oleracea* and *B. napus*—we performed a direct lineage homology analysis on the OrthoVenn2 web platform. The direct lineage clusters of the five species identified are presented in Figure 6, with 178 *WAK* proteins in the five Cruciferous species clustered into three homology groups. In total, 11 *B. juncea WAK*s, 2 *A. halleri WAK*s, 2 *A. lyrate WAK*s, 2 *A. alpina WAK*s, 10 *BnWAK*s, 7 *B. oleracea WAK*s, 4 *C. sativa WAK*s and 2 *B. rapa WAK*s did not cluster in any direct lineage cluster and were identified as singletons. 

In addition, as exhibited in Figure 6a between four Brassica plants, there were five core gene clusters (31 *WAK*s) among the four species, one gene cluster (4 *WAK*s) between *B. juncea* and *B. napus*, two gene clusters (4 *WAK*s) between *B. rapa* and *B. napus*, six gene clusters (12 *WAK*s) between *B. oleracea* and *B. napus* and in total we identified one Brassica-specific gene cluster (13 *WAK*s) among the three Brassica species. The absence of *B. rapa*- and *B. oleracea*-specific gene clusters in *B. napus* suggests that gene loss occurs during hybridization. Additionally, Figure 6d presents the relationship among *B. napus, B. rapa*, *B. oleracea* and *B. juncea*, especially between *B. napus* and *B. oleracea*.

*B. napus* was compared with four Arabidopsis plants and *C. sativa*. As shown in Figure 6b, there were three core gene clusters among those plants. It is worthwhile to mention that the Arabidopsis plants have no specific core gene clusters, except for *C. sativa* and *B. napus*, which have specific gene clusters. This result demonstrates that the Cruciferous plants have retained their affinities in evolution while also having evolutionary specificity among the subfamilies. A detailed list of direct gene clusters and single examples of gene clusters is given in Table S2.

### 2.7. Expression Pattern of BnWAK Gene in Response to Different Stresses

**Figure 7:**
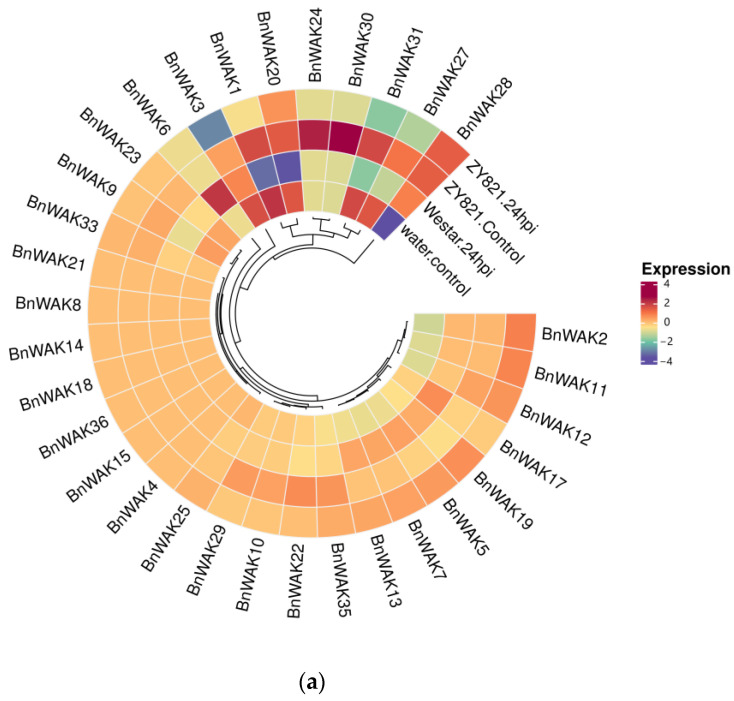

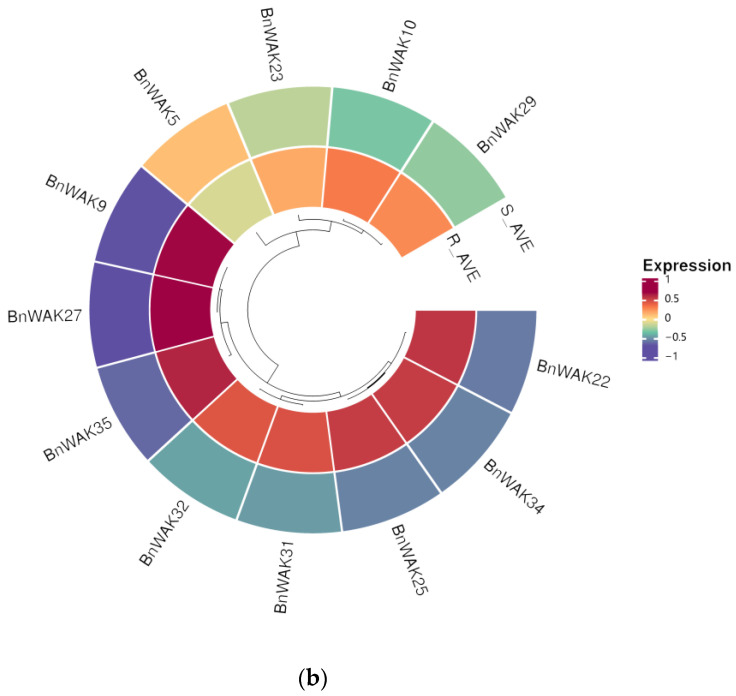
Analysis of *BnWAK* genes’ expression in *B. napus* leaves under biotic stress. (**a**). Expression levels of genome-wide *WAK* genes in both susceptible (Westar) and tolerant (ZY821) genotypes of *B. napus* leaves infected with *Sclerotinia sclerotiorum*. (**b**). Expression levels of genome-wide *WAK* genes in both susceptible (Zhongshuang11) and tolerant (White flower) genotypes of *B. napus* leaves infected with *Erysiphe cruciferarum*.

Based on transcriptome data of *B. napus* under different biotic stresses published on NCBI, the expression of *BnWAK*s under different biotic stresses was analyzed. The results showed that *BnWAK*s expressed differently under different stress treatments (Figure 7).

An analysis of transcriptome data from two varieties of *B. napus*, tolerant and susceptible, revealed significant differences in the expression of some *WAK* proteins between the two varieties following *Sclerotinia sclerotiorum* infection. The susceptible varieties *BnWAK24, 30* and *31* showed some increase after infection with *S. sclerotiorum*, while *BnWAK2*, *11, 19* and *5* showed some decrease after infection. Among the tolerant species, *BnWAK1*, *3*, *9*, *20*, *23*, *3*1 and *27* showed some increase, while *BnWAK2*, *28, 11* and *12* showed some decrease, especially *BnWAK28* (Figure 7a). Not coincidentally, powdery mildew caused with Cruciferous powdery mildew is an epidemic disease of *B. napus* growing worldwide. An analysis of the foliar transcriptome of immune varieties ‘White Flower’ and susceptible varieties ‘Zhong Shuang 11’ showed the highest expression of *BnWAK5* in the immune variety and *BnWAK35*, *32*, *25*, *29* and *10* in the susceptible variety (Figure 7b).

**Figure 8:**
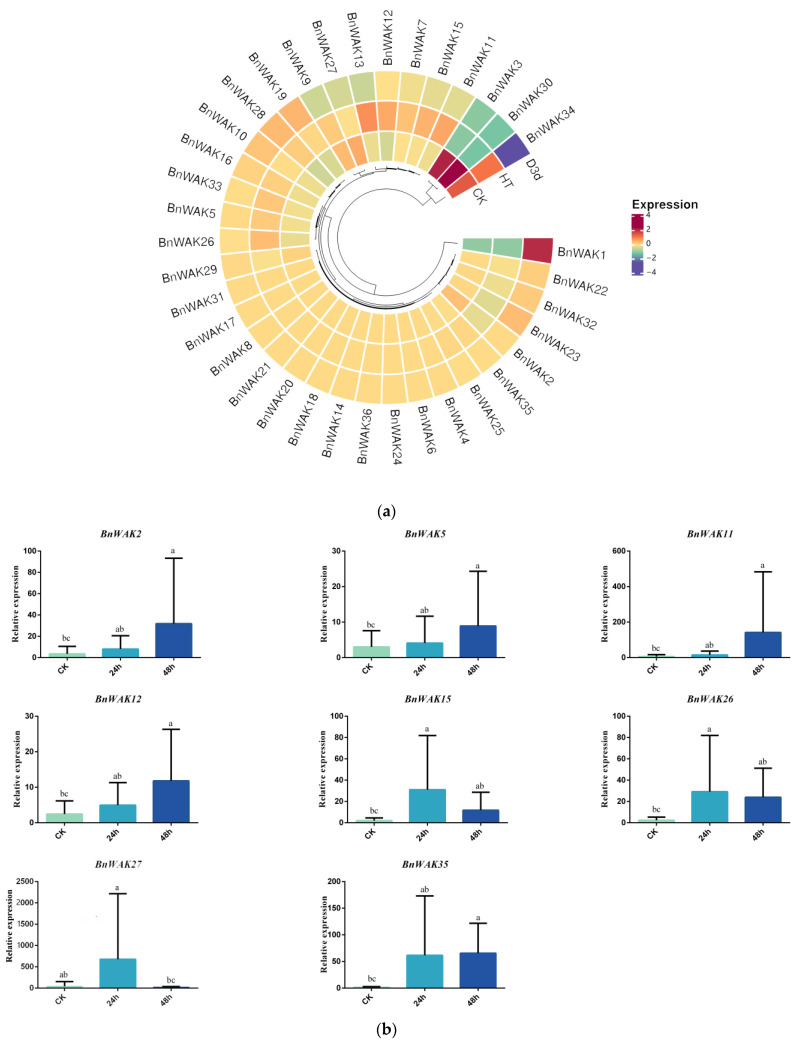
Analysis of *BnWAK* genes’ expression in *B. napus* under abiotic stress. (**a**). Expression of genome-wide *BnWAK* genes in *B. napus* leaves under drought and heat treatment. (**b**). qRT-PCR was used to measure drought-induced expression of selected *BnWAK* genes. Final results are exhibited as mean ± standard deviation. The labels 0 h (CK), 24 h and 48 h indicate the specific time points (in hours) at which the samples were collected for expression analysis following the stress treatment. LSD test was employed to determine significant differences among them, where distinct letters indicate a significant difference (*p* < 0.05).

The *WAK* family also has an active expression during the abiotic stress response. As the frequency of heat and droughts increases with global warming, the analysis of transcriptome data from *B. napus* under heat and drought stresses showed that the expression of *BnWAK34*, *13* and *12* increased to some extent under heat treatment, with *BnWAK34* being the most significant. The expression of *BnWAK1* and *23* was downregulated. The situation was different under drought treatment, where the expression of *BnWAK3* and *30* was increased (Figure 8). This suggests that the *BnWAK* genes are also closely related to the response to a high temperature and drought.

### 2.8. Expression Pattern of BnWAK Gene in Oil Accumulation

**Figure 9:**
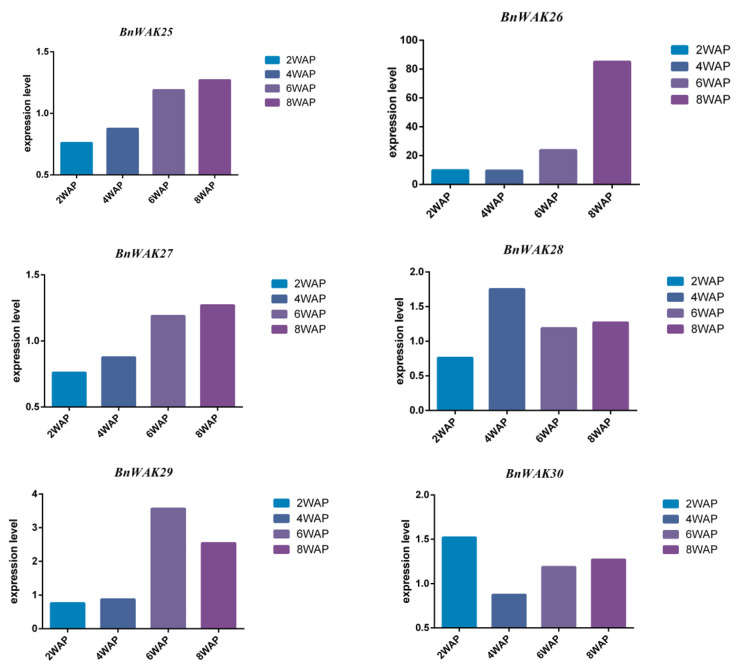

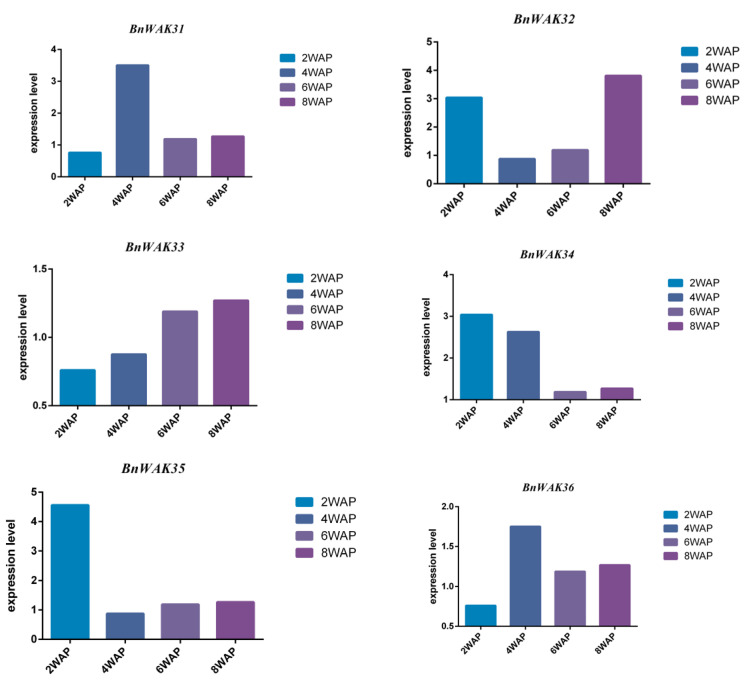
Expression analysis of *BnWAK* genes during fatty acid biosynthesis. Expression levels of genome-wide *WAK* genes in developing seeds of *B. napus* at 2, 4, 6 and 8 weeks after pollination.

All *BnWAK* genes were analyzed using an RNA-seq analysis at 2, 4, 6 and 8 weeks after pollination to determine their potential functions during fatty acid biosynthesis. The results indicated that 36 *BnWAK* genes tended to change their expression during fatty acid biosynthesis (Figure 9). Especially, the expression of *BnWAK6, 7* and *12* were significantly upregulated between 2 and 4 weeks and dramatically downregulated after 4 weeks after pollination, which exhibited a tight relationship with the biosynthesis of fatty acids. In contrast, the expression of *BnWAK10*, *26* and *29* was at a low level between 2 and 4 weeks, but showed a significant raise after 6 weeks, which suggested the connection with the degradation of fatty acids. After pollination, the expression of several genes, such as *BnWAK4*, *9*, *14, 18*, 20, 25, 27 and 33, was always upregulated, indicating that these genes may be related to seed development. As a whole, different *BnWAK* genes exhibited different expression patterns during fatty acid biosynthesis, even duplicated genes.

## 3. Discussion

Plants are capable of responding to various stresses and can modulate various developmental processes, including photomorphogenesis and circadian rhythms [36]. *WAK* genes have been demonstrated to play an imperative role in the response pathway of plants sensing pathogen invasion to regulate cell expansion [10,34]. In this study, a total of 178 *WAK* genes were identified from nine Cruciferous species including *A. thaliana, A. lyrate, A. helleri, A. alpina, C. sativa, B. juncea, B. napus, B. rapa* and *B. oleracea*. Previous studies have found 130 members of the *WAK* gene family in rice [37], 23 in roses [38], 29 in cotton [16,17] and 11 in walnuts [6].

A phylogenetic analysis of *WAK* family genes revealed that the number of *WAK* genes varied greatly among Cruciferous species, and the expansion of the *WAK* genes followed the duplication of the genome (Figure 1). Furthermore, the evolution of *WAK* genes displayed a clear species bias, with the duplication of species genes occurring after the divergence of species. According to the theory of the triangle of U, *B. napus* was derived from natural interspecific crosses between kale and cabbage [39]. For example, the genome size of *B. napus* was nearly twice that of *B. rapa* and *B. oleracea*, which may have led to the numbers of *WAK* members in *B. napus* being nearly twice those of *B. rapa* and *B. oleracea*. However, the *WAK* numbers in the Arabidopsis are not much different.

Evidence for the evolution of species or genes can be found in the gene structure and motif distribution (Figure 2). Accordingly, members of the same subfamily exhibited consistent exon/intron and motif distribution patterns, suggesting a similar role in functions [40,41]. According to Figure 2b, the classification based on the phylogenetic tree was further confirmed with the gene structure and motif distribution analysis. Furthermore, the structure and number of introns of the *WAK* genes were well conserved among the nine Cruciferous species. This is consistent with previous studies [18]. The number of thirty-six *BnWAK* genes on chromosomes ranged from one to eight, and most of these genes were randomly distributed on chromosomes C02, C03, C05, C06, C08, A06, A07, A08 and A09, with five genes unable to be localized on chromosomes. Subcellular localization predictions for the *BnWAK* gene family revealed that most of the genes in this gene family were localized to be expressed in the extracellular space, suggesting that the *BnWAK* family is important for the plant immunity of *B. napus*.

In response to a variety of stresses, cis-elements contribute to regulate and function certain genes [40,42]. The cis-acting element analysis revealed that *BnWAK* genes contain a significant number of elements with various functions (Figure 4). As a result, *BnWAK*s may play an influential role in the tolerance to environmental stress and the response to light during the life of plants. Evidence indicates that *WAK* genes contribute to plant immunity in multiple ways, including defense against pathogens with diverse lifestyles [3]. This may partly explain why *BnWAK* promoter regions contain so many cis-elements (Figure 4). It remains to be determined how the *BnWAK* genes regulate different stress-responsive functions as cis-elements exist in the upstream sequences.

The growth and yield of *B. napus* can be significantly affected by both biotic and abiotic stresses [33], such as heat, droughts and pathogen infection. Previous studies have demonstrated that *WAK* is a critical member for the disease resistance of fungus and bacteria in several cereal crop species. There have been extensive studies of *WAK* in several plant species under varying stresses, including rice [15,43,44], maize [13,14] and blue carpet [45]. In rice, *WAK* genes are not only responsible for the somatic regulation of elongation growth [46] but also play an instrumental role in resistance to various stresses [14,15]. For instance, *OsWAK11* is the link between changes in cell wall pectin methylation and the Brassinosteroid (BR) signaling pathway, which together regulate adaptive changes in the cell growth rate [43]. *OsWAK14*, *OsWAK91* and *OsWAK92* are important members of the rice blast resistance [44]. For maize, the maize *WAK* gene *ZmWAK-RLK1 (Htn1)* is involved in resistance to northern corn leaf blight (NCLB) by affecting benzoxazin (BX) secondary metabolites [43]. Not coincidentally, *ZmWAK (qHSR1)* prevented the endophytic nutritional growth of *S. reilianum* and conferred resistance to maize silky black ears disease [13]. It was also observed that *B. napus WAK* genes were expressed under various stresses in our study. The expression levels of these genes differed under different stresses, indicating that they were widely expressed under different conditions. The results of our study indicated that several genes are associated with resistance to *S. sclerotiorum* infection, including *BnWAK28*, *BnWAK6*, *BnWAK24* and *BnWAK30*. Moreover, *BnWAK5* appears to contribute to resistance to powdery mildew. Furthermore, heat stress increased the expression of *BnWAK34*, *13* and *12*, and inhibited the expression of *BnWAK1* and *23*. Additionally, drought treatment induced the expression of *BnWAK3* and *30* (Figure 8). The results indicate that *BnWAK* genes have an influential role in the biotic and abiotic stress response.

Furthermore, as an oilseed crop, it is crucial to increase oil production or alter fatty acid fractions [47]. And fatty acid biosynthesis occurs mainly between 2 and 4 weeks after pollination, while degradation occurs mainly after 6 weeks [47]. Additionally, we examined the expression of *BnWAK*s during different stages of fatty acid biosynthesis. Interestingly, we found that *BnWAK*s tend to change over time (Figure 9). A significant increase in the expression of several genes was observed during the fatty acid synthesis stage, including *BnWAK2*, *BnWAK5*, *BnWAK7* and *BnWAK12*. In light of these findings, it appears that genes in the *BnWAK* family may have a role in fatty acid biosynthesis. In future work, stress-related *WAK* genes associated with fatty acid synthesis in *B. napus* will be used to breed more stress-resistant and high-yielding varieties, and to explore the mechanisms behind these genes. In future work, stress-related *WAK* genes and fatty acid synthesis in *B. napus* will be used to breed more stress-tolerant and high-yielding varieties and to explore the stress tolerance mechanisms behind these genes.

## 4. Materials and Methods

### 4.1. Growth Conditions and Plant Materials

*B. napus* (Zheda 630) seeds were collected from the Institute of Crop Science and Zhejiang Key Laboratory of Crop Germplasm, Zhejiang University, Hangzhou, China. The seeds were germinated in a saturated seedling sponge and subsequently placed in a light-deprived environment, ensuring their constant moisture. After the 4-day germination phase, *B. napus* seedlings were transplanted into a hydroponic tank filled with a Hoagland nutrient solution, maintained under controlled conditions of 24/20 °C (day/night), a photoperiod of 14/10 h (light/night) and a relative humidity of 60–70%. The seedlings were exposed to an active photon flux below 200 μmol m^−2^ s^−1^ [48]. Drought treatment was inflicted by applying the commonly employed 15% PEG6000 solution. After 35 days, *B. napus* seedlings were stressed with 15% PEG6000 at 24 h and 48 h. There were three treatments in total, i.e., CK (0 h), 24 hours (24 h) and 48 hours (48 h).

### 4.2. Identification and Analysis of WAK Family Members in Nine Cruciferous Species

Data from Ensemble (http://plants.ensembl.org/index.html (accessed on 1 January 2022) were obtained for *A. thaliana*, *A. lyrata*, *A. halleri*, *A. alpina*, *C. sativa*, *B. juncea*, *B. napus*, *B. rapa* and *B. oleracea* genomes. From the Pfam database, the term *WAK*-domain search model accession (PF08488) [46] was used to identify potential *WAK* genes with the e-value set to 10^−3^ as the criterion (https://www.ebi.ac.uk/Tools/hmmer/ (accessed on 1 January 2022)) [49,50]. To identify members of the *WAK* gene family, candidate sequences were searched in the SMART and NCBI CDD databases [51,52,53].

### 4.3. Alignment of Multiple Sequences and Construction of Phylogenetic Trees

A phylogenetic analysis of the *WAK* protein family in nine Cruciferous species was performed. The *WAK* protein sequences identified from other species and *B. napus* were compared across multiple sequences using Clustal W available in MEGA11 [54,55]. A maximum likelihood phylogenetic tree was constructed with MEGA11 for the *WAK* from nine plants, with a bootstrap value of 1000. This tree was used as the optimal model after computational simulation.

### 4.4. Genomic Information and Analysis of Physiological and Biochemical Properties

Based on obtained *BnWAK* gene sequence information, *BnWAK*s were localized to their corresponding positions on chromosomes using TBtools [56,57]. Information on the theoretical isoelectric point, molecular mass, amino acid length and other characteristics of *BnWAK*s was analyzed and predicted using the ExPASy website (https://www.expasy.org/ (accessed on 2 January 2022)) [58,59]. Using the WoLF PSORT (http://wolfpsort.hgc.jp/ (accessed on 2 January 2022)) and CELLO v2.5 (http://cello.life.nctu.edu.tw/ (accessed on 2 January 2022)) online tools, the subcellular localization was analyzed [60,61,62].

### 4.5. Analysis of WAK Family Protein Sequences for Gene Structure, Motifs and Gene Promoter Cis-Acting Elements

MEME was used to analyze conserved motifs in the *WAK* gene family (https://meme-suite.org/meme/ (accessed on 3 January 2022)) [40,59]. Perl scripts were used to obtain information related to the gene structure, such as exons and introns, from GFF files. Data visualization was performed using TBtools [53,57]. A Perl script was utilized to extract the 1500 bp DNA sequences upstream of the identified *WAK* genes. To identify cis-regulatory components (CREs) and select those involved in abiotic and hormonal stress, the Plant CARE database (http://bioinformatics.psb.ugent.be/webtools/plantcare/html/ (accessed on 4 January 2022)) was utilized.

### 4.6. Collinearity Analysis and Homologous Gene Analysis

The collinearity analysis was performed on the genomes of *B. napus* with *A. thaliana*, *A. halleri*, *A. lyrate*, *A. alpina*, *C. sativa*, *B. juncea*, *B. rapa*, *B. oleracea* and *B. carinata* using McscanX [61,62] and mapped using TBtools [62] software. The direct homologous gene analysis was performed using OrthoVenn2 (https://orthovenn2.bioinfotoolkits.net/home (accessed on 4 January 2022)) on *B. napus* with *B. juncea, B. rapa* and *B. oleracea* [63].

### 4.7. Expression and Stress Response Expression Profiling of BnWAK

To investigate expression (under stress and non-stressed conditions) of the *BnWAK*s, RNA-Seq data were downloaded from previously published data that were downloaded from the NCBI database (GSE77637, GSE81545, GSE156029 and GSE188377) [34,64,65]. GSE77637 was analyzed to investigate the expression of *BnWAK*s from *B. napus* at different developmental stages [31]. GSE81545 was used to explore the expression of *BnWAK*s from *B. napus* after *Sclerotinia sclerotiorum* infection [34]. GSE188377 was used to investigate the expression of *BnWAK*s from *B. napus* after *Erysiphe cruciferarum* infection [65]. GSE156029 was applied to explore *BnWAK* expression under a drought and heat stress. After log10 (FPKM + 1 × 10^–5^) normalization, heatmaps were generated with OmicStudio tools (https://www.omicstudio.cn (accessed on 7 January 2022)) [66,67].

The total RNA of *B. napus* (Zheda 630) under 15% PEG6000 treatment at different times was isolated with a FastPure Plant Total RNA Isolation Kit (Vazyme, Nanjing, China). Zero hours was used as a reference, *ACT2* was used as the internal reference gene and Primer-BLAST (https://blast.ncbi.nlm.nih.gov/Blast.cgi (accessed on 7 January 2022)) was used to design the primers. The relevant primers are shown in Table S3. Six samples were taken at each time point. Three replicates of each sample were presented using ChamQ Universal SYBR qPCR Master Mix (Vazyme). The reaction conditions are according to the instructions of ChamQ Universal SYBR qPCR Master Mix (Vazyme); more information is presented in Table S4. The 2^−ΔΔCt^ method was used to analyze the results [68]. The qRT-PCR analysis involved eight selected *BnWAK*s, including *BnWAK2*, *BnWAK5*, *BnWAK11*, *BnWAK12, BnWAK15*, *BnWAK26*, *BnWAK27* and *BnWAK35*.

## Data Availability

Data available on request from the authors.

## Supplementary Materials

The following supporting information can be downloaded at: https://www.mdpi.com/article/10.3390/ijms241713601/s1.
