# Supplementary material for: Analysis of WAK Genes in Nine Cruciferous Species with a Focus on Brassica napus L
Source: Int J Mol Sci. 2023 Sep 2;24(17):13601. doi: 10.3390/ijms241713601 (PMC10487794; doi:10.3390/ijms241713601)
Supplement: Supplementary file 1 [file ijms-24-13601-s001.zip › ijms-2523488-supplementary.pdf]

Table S1. a Information of WAK genes identified in Brassica napus.

No.	Gene ID	Name	Chr	Start	End	Strand	Gene length (bp)	MW (Da)	pI	Protein length (aa)	Predicted subcellular localization
1	BnaA07g20220D	BnWAK29	A07	3190904	3193544	+	2640	88827.6	6.54	794	Extracellular
2	BnaC01g07330D	BnWAK18	C01	931665	932699	-	1034	37299.9	4.96	336	Extracellular
3	BnaC01g07320D	BnWAK14	C01	960253	962795	+	2542	53346.8	6.49	488	Extracellular
4	BnaC07g43130D	BnWAK19	C07	485653	488138	+	2485	79137.4	5.72	708	Extracellular
5	BnaC06g19690D	BnWAK32	C06	1421046	1423329	-	2283	77729.3	6.88	695	Extracellular
6	BnaC06g19670D	BnWAK22	C06	1442279	1448773	-	6494	88367.4	6.5	790	Extracellular
7	BnaC08g38530D	BnWAK10	C08	611740	614043	+	2303	79491.2	8.17	713	Extracellular
8	BnaC08g38510D	BnWAK3	C08	617501	619780	+	2279	61235.6	6.08	554	Extracellular
9	BnaC08g38450D	BnWAK23	C08	630820	632703	-	1883	64406.9	6.55	576	Extracellular
10	BnaC08g18820D	BnWAK17	C08	304942	306614	+	1672	50804	6.5	452	Extracellular
11	BnaC08g18800D	BnWAK31	C08	312330	318041	+	5711	72286.4	5.99	659	Extracellular
12	BnaC07g07440D	BnWAK21	C07	1557507	1558826	-	1319	23712	7.75	216	Extracellular
13	BnaC08g01050D	BnWAK8	C08	1003264	1005111	+	1847	41931.7	6.47	372	Extracellular
14	BnaC08g01030D	BnWAK1	C08	1015241	1017749	+	2508	82486	7.64	743	Extracellular
15	BnaA07g35110D	BnWAK16	A07	289545	291209	+	1664	41462.3	4.8	382	Extracellular
16	BnaA07g35080D	BnWAK13	A07	292735	294757	+	2022	65751.7	7.9	599	Extracellular
17	BnaC06g40010D	BnWAK11	C06	1630822	1633244	-	2422	84017	6.22	756	Extracellular
18	BnaC06g40020D	BnWAK35	C06	1633685	1636228	-	2543	85382.3	6.94	771	Extracellular
19	BnaA08g22050D	BnWAK9	A08	481624	485678	+	4054	87137.9	5.61	786	Extracellular
20	BnaA08g23630D	BnWAK33	A08	1087716	1089791	+	2075	63698.8	6.55	570	Extracellular
21	BnaA08g28110D	BnWAK24	A08	374630	377123	-	2493	72038.1	8.21	650	Extracellular
22	BnaA08g28120D	BnWAK7	A08	378356	380919	-	2563	83946.3	7.03	756	Extracellular
23	BnaA09g44180D	BnWAK36	A09	62165	63907	+	1742	27631.8	6.22	246	Extracellular
24	BnaA09g44200D	BnWAK27	A09	67143	73655	+	6512	80579.7	6.66	729	Extracellular
25	BnaC02g25920D	BnWAK28	C02	598805	601254	-	2449	77966	5.32	703	Extracellular
26	BnaC03g57020D	BnWAK25	C03	607800	612841	+	5041	75640.9	7.85	670	Extracellular
27	BnaA06g13520D	BnWAK26	A06	44185	45691	+	1506	37041.9	9.41	338	Extracellular
28	BnaA01g06080D	BnWAK15	A01	426263	428868	-	2605	88241.9	6.15	787	Extracellular
29	BnaA01g06050D	BnWAK6	A01	442103	444612	+	2509	73345.7	5.99	667	Extracellular
30	BnaC05g12280D	BnWAK20	C05	224659	225887	+	1228	36122.4	5.84	333	Extracellular
31	BnaA08g20730D	BnWAK4	A08	155034	157964	-	2930	79837	7.87	715	Extracellular
32	BnaA09g56430D	BnWAK30	A09	21912	24167	-	2255	74912.7	7.52	677	Extracellular
33	BnaA09g56440D	BnWAK12	A09	24607	27027	-	2420	84229.2	7.23	752	Extracellular
34	BnaA09g56450D	BnWAK5	A09	27869	33406	-	5537	109667.6	7.91	990	Extracellular
35	BnaC05g49930D	BnWAK34	C05	7978	9273	-	1295	41888.3	7.76	379	Extracellular
36	BnaA06g38500D	BnWAK2	A06	321	4509	-	4188	70800.4	5.17	634	Extracellular

Table S1. b Information of WAK genes identified in Arabidopsis halleri.

No.	Name	Gene ID	Chr	Start	End	Strand	Gene length (bp)	Protein length (aa)	MW (Da)	pI
1	ArhWAK1	g01535	FJVB01000002.1	3053039	3058599	-	5560	1463	163202.2	6.43
2	ArhWAK2	g09983	FJVB01000023.1	592205	594115	-	1910	381	41676.8	6.8
3	ArhWAK3	g09981	FJVB01000023.1	586093	588605	-	2512	681	76048.5	7.65
4	ArhWAK4	g09982	FJVB01000023.1	589189	591715	-	2526	715	79524.5	8.56
5	ArhWAK5	g20093	FJVB01000084.1	558053	560911	-	2858	794	88767.3	6.97
6	ArhWAK6	g25902	FJVB01000164.1	157628	160381	-	2753	751	83969.1	7.32
7	ArhWAK7	g03700	FJVB01000006.1	1670	4601	-	2931	783	87507.7	6.74
8	ArhWAK8	g13734	FJVB01000037.1	801001	804233	+	3232	739	82011.8	6.46
9	ArhWAK9	g26929	FJVB01000187.1	65478	68371	-	2893	786	87774.2	6.73
10	ArhWAK10	g09979	FJVB01000023.1	579328	582045	-	2717	710	78425.8	7.04
11	ArhWAK11	g09980	FJVB01000023.1	582904	585545	-	2641	761	84488.5	6.27
12	ArhWAK12	g00882	FJVB01000001.1	4105235	4107654	-	2419	715	79524.5	8.56
13	ArhWAK13	g30144	FJVB01000324.1	87713	91091	-	3378	730	80710	8.5
14	ArhWAK14	g27507	FJVB01000202.1	246861	250358	+	3497	689	77883	5.85
15	ArhWAK15	g31300	FJVB01000519.1	14216	19361	-	5145	755	84547.9	6.17
16	ArhWAK16	g09970	FJVB01000023.1	557580	560121	+	2541	735	82648.7	6.46

Table S1. c Information of WAK genes identified in Arabidopsis lyrata.

No.	Gene ID	Name	Chr	Start	End	Strand	Gene length (bp)	Protein length (aa)	MW (Da)	pI
1	fgenesh2_kg.7__1069__AT4G31110.1	AlyWAK1	7	4477428	4480284	-	2856	792	88923.4	5.78
2	scaffold_302957.1	AlyWAK2	3	11559165	11562318	-	3153	730	80770.1	8.56
3	scaffold_304266.1	AlyWAK3	3	23250139	23252754	+	2615	596	66109.6	6.97
4	fgenesh2_kg.1__1765__AT1G16090.1	AlyWAK4	1	6785802	6787332	+	1530	322	35778.5	6.47
5	scaffold_102165.1	AlyWAK5	1	8204722	8207347	-	2625	791	88215.5	6.78
6	Al_scaffold_0001_1675	AlyWAK6	1	6797719	6800353	+	2634	682	75360.6	7.54
7	fgenesh1_pm.C_scaffold_1001383	AlyWAK7	1	6822060	6824448	-	2388	735	82558.7	6.79
8	Al_scaffold_0003_3888	AlyWAK8	3	23238170	23240737	+	2567	652	72610	6.56
9	fgenesh2_kg.1__1959__AT1G17910.1	AlyWAK9	1	7518155	7520696	+	2541	737	82489.6	7.76
10	fgenesh2_kg.2__1234__AT1G69730.1	AlyWAK10	2	13933346	13936005	-	2659	768	86329.5	7.26
11	Al_scaffold_0001_1673	AlyWAK11	1	6791159	6793506	+	2347	680	75448.6	7.69
12	fgenesh2_kg.1__1768__AT1G16150.1	AlyWAK12	1	6794228	6796666	+	2438	773	86488.9	6.22
13	Al_scaffold_0001_3546	AlyWAK13	1	16499412	16502915	+	3503	708	80044.6	6.86
14	fgenesh2_kg.1__1766__AT1G16110.1	AlyWAK14	1	6787946	6790332	+	2386	629	70012.3	7.79

Table S1. d Information of WAK genes identified in Arabidopsis thaliana.

No.	Name	Gene ID	Chr	Start	End	Strand	Gene length (bp)	Protein length (aa)	MW (Da)	pI
1	AtWAK1	AT1G16110	1	5518219	5520929	+	2710	642	70816.4	8.25
2	AtWAK2	AT1G16130	1	5525293	5528212	+	2919	748	83356.7	6.72
3	AtWAK3	AT1G79670	1	29976725	29979356	-	2631	751	83383.5	6.5
4	AtWAK4	AT1G19390	1	6700756	6703368		2612	788	87390.6	7.63
5	AtWAK5	AT1G16150	1	5532298	5535060	+	2762	761	84669.6	5.65
6	AtWAK6	AT1G16260	1	5559498	5562192	-	2694	720	81159.6	8.03
7	AtWAK7	AT1G16160	1	5535766	5538501	+	2735	731	80739.8	7.4
8	AtWAK8	AT1G16090	1	5516259	5517822	+	1563	317	34428.2	6.49
9	AtWAK9	AT1G69730	1	26228565	26231375	-	2810	792	88595.3	7.74
10	AtWAK10	AT4G31110	4	15127045	15130021	+	2976	793	88753.3	5.99
11	AtWAK11	AT1G16120	1	5522602	5525452	+	2850	730	81209.8	6.7
12	AtWAK12	AT1G17910	1	6159126	6161615	+	2489	764	85211.1	6.97
13	AtWAK13	AT1G79680	1	29979973	29982819	-	2846	769	85703.2	5.72
14	AtWAK14	AT4G31100	4	15123763	15126884	+	3121	786	87303.7	6.4

Table S1. e Information of WAK genes identified in Arabis alpina.

Gene ID	Name	Gene ID	Chr	Start	End	Strand	Gene length (bp)	Protein length (aa)	MW (Da)	pI
KFK28618	AalWAK1	AALP_AA7G022800	7	1965407	1968528	-	3121	687	76622.6	7.87
KFK28619	AalWAK3	AALP_AA7G023000	7	1973172	1975578	-	2406	708	78525.2	7.21
KFK29692	AalWAK2	AALP_AA7G166300	7	20201963	20204687	+	2724	728	81090.1	7.45
KFK42836	AalWAK7	AALP_AA1G045300	1	2199225	2200945	-	1720	494	55034.3	8.67
KFK42837	AalWAK5	AALP_AA1G045500	1	2205018	2208395	-	3377	563	62946.3	8.24
KFK43950	AalWAK4	AALP_AA1G195700	1	13051190	13053659	-	2469	695	77717.7	6.75
KFK44073	AalWAK6	AALP_AA1G212600	1	14651527	14653547	-	2020	556	62341.9	5

Table S1. f Information of WAK genes identified in Brassica juncea.

Name	Gene ID	Chr	Start	End	Strand	Gene length (bp)	Protein length (aa)	MW (Da)	pI
BjuWAK1	BjuB03g37070S	B03	48938967	48940222	+	1255	299	33493.3	8.22
BjuWAK2	BjuB03g58320S	B03	60749760	60754475	+	4715	762	84784.7	6.55
BjuWAK3	BjuA02g19030S	A02	18421289	18421805	+	516	145	15658.6	4.64
BjuWAK4	BjuB03g35610S	B03	48024855	48027716	+	2861	782	86103.7	6.9
BjuWAK5	BjuB03g52050S	B03	57906781	57909550	+	2769	782	87256.2	6.72
BjuWAK6	BjuB03g58330S	B03	60755939	60770870	+	14931	447	50290.1	4.94
BjuWAK7	BjuB08g12460S	B08	10369471	10373872	+	4401	726	82333.6	7.68
BjuWAK8	BjuB05g56710S	B05	62300206	62303762	+	3556	483	53353.4	7.79
BjuWAK9	BjuA09g07560S	A09	4090080	4097587	+	7507	937	102542.8	8.14
BjuWAK10	BjuB06g14420S	B06	9901784	9904818	+	3034	792	88210.4	7.32
BjuWAK11	BjuB03g52040S	B03	57901577	57904636	+	3059	801	89601.2	6.4
BjuWAK12	BjuA01g37990S	A01	31489259	31495408	+	6149	386	42829.4	8.59
BjuWAK13	BjuB03g37130S	B03	48951849	48959012	+	7163	503	54956.8	6.56
BjuWAK14	BjuB03g53440S	B03	58542726	58545466	+	2740	721	80525.2	6.61
BjuWAK15	BjuB03g35590S	B03	48021472	48022278	+	806	268	28970.1	4.51
BjuWAK16	BjuA09g07570S	A09	4098175	4099251	+	1076	294	32234.1	5.43
BjuWAK17	BjuB04g25730S	B04	39208997	39211730	+	2733	712	79943	5.31
BjuWAK18	BjuB05g56720S	B05	62304223	62308417	+	4194	488	53383.8	6.17
BjuWAK19	BjuB06g14410S	B06	9889757	9898462	+	8705	753	84174.8	6.28

Table S1. g Information of WAK genes identified in Brassica oleracea.

Name	Gene ID	Chr	Start	End	Strand	Gene length (bp)	Protein length (aa)	MW (Da)	pI
BoWAK19	Bo1g011590	C1	4136640	4139097	-	2457	510	56503	6.55
BoWAK5	Bo1g011610	C1	4146254	4148744	-	2490	565	62641.9	5.04
BoWAK4	Bo1g011630	C1	4157579	4161164	-	3585	845	93942.4	6.46
BoWAK2	Bo1g011640	C1	4167991	4170657	+	2666	785	87494.8	6.02
BoWAK10	Bo2g095480	C2	26834996	26837477	-	2481	760	84765.5	6.95
BoWAK7	Bo2g095500	C2	26840111	26842548	-	2437	582	64403.7	5.14
BoWAK24	Bo3g055310	C3	21846648	21859989	+	13341	2408	269123	7.72
BoWAK26	Bo3g134650	C3	49530527	49533233	-	2706	629	71154.8	6.96
BoWAK16	Bo5g021560	C5	7686417	7689768	-	3351	580	64984.1	6.12
BoWAK18	Bo5g021580	C5	7700621	7701846	-	1225	332	35898.1	5.83
BoWAK23	Bo5g027440	C5	9577898	9579701	-	1803	261	28970.1	6.09
BoWAK15	Bo5g027450	C5	9587265	9592704	-	5439	883	98878.6	5.28
BoWAK6	Bo6g080830	C6	24760290	24762931	+	2641	794	88707.4	6.88
BoWAK11	Bo6g080850	C6	24769095	24773845	+	4750	773	86643.2	6.35
BoWAK22	Bo6g124050	C6	39445521	39447090	-	1569	472	51599.6	8.26
BoWAK8	Bo6g124060	C6	39447535	39450102	-	2567	779	86143.2	6.82
BoWAK17	Bo7g114950	C7	45748529	45751030	+	2501	752	84673.2	6.65
BoWAK12	Bo8g004310	C8	830018	832521	-	2503	743	82449	7.42
BoWAK25	Bo8g004320	C8	844071	846557	-	2486	619	69843.9	7.8
BoWAK1	Bo8g066660	C8	21431261	21433565	-	2304	719	80650.8	7.32
BoWAK3	Bo8g068520	C8	22475966	22478117	-	2151	612	68087.1	7.18
BoWAK14	Bo8g102730	C8	36230328	36231077	+	749	249	26792.7	4.77
BoWAK21	Bo8g105580	C8	37218165	37220510	+	2345	691	77788.1	6.95
BoWAK13	Bo8g105630	C8	37231770	37233971	-	2201	536	58970.5	7.54
BoWAK9	Bo8g105640	C8	37234407	37236832	-	2425	575	63986.7	5.84
BoWAK20	Bo8g105650	C8	37237374	37239677	-	2303	546	60472	7.07

Table S1. h Information of WAK genes identified in Brassica rapa.

Name	Gene ID	Chr	Start	End	Strand	Gene length (bp)	Protein length (aa)	MW (Da)	pI
BrWAK1	Bra003598	A07	13985750	13988380	+	2630	791	88173.9	6.65
BrWAK2	Bra030596	A08	21165894	21168474	+	2580	750	83178.4	7.57
BrWAK3	Bra011247	A01	3096953	3099501	-	2548	725	80259.5	6.61
BrWAK4	Bra026731	A09	33429683	33431938	-	2255	725	80078.9	7.86
BrWAK5	Bra031079	A09	32557294	32563071	+	5777	907	100190.7	7.72
BrWAK6	Bra016370	A08	18085794	18089327	-	3533	710	79282.4	8.03
BrWAK7	Bra011245	A01	3114082	3116687	+	2605	787	88117.6	6.05
BrWAK8	Bra026732	A09	33432378	33434806	-	2428	754	84120.9	6.74
BrWAK9	Bra030595	A08	21169709	21172220	+	2511	701	77694.7	7.9
BrWAK10	Bra018131	A06	10480033	10482794	+	2761	390	43691.8	8.22
BrWAK11	Bra025736	A06	7581494	7586315	-	4821	962	107772.7	6.67
BrWAK12	Bra015361	A10	2272350	2275411	+	3061	792	88461.4	7.67
BrWAK13	Bra035134	A07	22313216	22315642	-	2426	752	83375.4	6.44

Table S1. i Information of WAK genes identified in Camelina satica.

Name	Gene ID	Chr	Start	End	Strand	Gene length (bp)	Protein length (aa)	MW (Da)	pI
CsaWAK14	Csa01g034720	1	16386228	16389318	-	3090	802	88782	6.65
CsaWAK3	Csa03g019890	3	7274452	7292674	+	18222	3758	416011.2	7.17
CsaWAK13	Csa03g019980	3	7315048	7317573	-	2525	736	82625.7	6.95
CsaWAK30	Csa03g021720	3	8064764	8066147	+	1383	384	42094	6.23
CsaWAK9	Csa03g036960	3	16261670	16266531	+	4861	742	82287.3	7.87
CsaWAK11	Csa05g087560	5	31358922	31362118	-	3196	883	97595.8	7.3
CsaWAK33	Csa06041s010	unpScaffold06041	16	522	-	506	214	240688	6.6
CsaWAK26	Csa07g036470	7	19795571	19798411	-	2840	799	89075.4	6.95
CsaWAK4	Csa07g058680	7	29764157	29766673	+	2516	615	69053.7	5.19
CsaWAK32	Csa08449s010	unpScaffold08449	3	368	-	365	595	67735.4	8.15
CsaWAK10	Csa09g093940	9	35466954	35477475	-	10521	1627	180537	6.53
CsaWAK12	Csa10g011400	10	4559418	4562127	-	2709	799	89223.5	6.44
CsaWAK27	Csa11g012260	11	5107397	5110297	-	2900	804	89630.2	7.41
CsaWAK5	Csa12g016000	12	4812010	4817607	-	5597	796	88911.4	6.75
CsaWAK17	Csa14g020220	14	7663155	7665243	+	2088	431	47454.7	7.31
CsaWAK31	Csa14g020230	14	7666320	7668989	+	2669	520	57893.3	6.31
CsaWAK6	Csa14g020240	14	7670198	7672614	+	2416	750	83939.2	8.19
CsaWAK18	Csa14g020250	14	7673356	7675713	+	2357	734	81916.7	6.85
CsaWAK28	Csa14g020260	14	7676475	7678914	+	2439	758	84591.8	6.64
CsaWAK22	Csa14g020270	14	7679565	7681615	+	2050	604	66688.8	6.5
CsaWAK21	Csa14g020350	14	7709763	7712100	-	2337	720	81127.1	7.49
CsaWAK1	Csa14g022240	14	8544240	8546768	+	2528	690	76994.6	6.1
CsaWAK23	Csa14g042590	14	17910380	17913552	+	3172	702	78276.7	7.07
CsaWAK2	Csa16g031260	16	16630373	16632813	-	2440	739	82001.1	6.64
CsaWAK20	Csa16g049330	16	25398052	25400984	-	2932	833	92640	7.75
CsaWAK24	Csa17g021680	17	7418900	7420754	+	1854	441	48629.2	7.85
CsaWAK8	Csa17g021690	17	7421388	7427090	+	5702	1041	115283.4	5.62
CsaWAK15	Csa17g021700	17	7427674	7433111	+	5437	1446	160961	7.06
CsaWAK25	Csa17g021720	17	7439459	7445574	+	6115	1591	176324.8	6.4
CsaWAK19	Csa17g021810	17	7473776	7476262	-	2486	717	80083.9	7.14
CsaWAK29	Csa17g023680	17	8262117	8264644	+	2527	758	84720.8	7.92
CsaWAK16	Csa17g051990	17	18944292	18947535	+	3243	715	79702	6.71
CsaWAK7	Csa19g050880	19	22236613	22239733	+	3120	791	87500.7	7.59

Table S2. Detailed list of orthologous gene clusters between the WAK genes in A. thaliana, A. lyrate, A. helleri, A. alpina, C. sativa, B. juncea, B. rapa, B. carinata, B oleracea and B. napus.

Cluster number						
1	AtWAK5	AtWAK2	CsaWAK28	CsaWAK25	CsaWAK15	
2	CsaWAK26	CsaWAK10	CsaWAK6	CsaWAK18	CsaWAK4	CsaWAK20
3	CsaWAK8	AalWAK1	BrWAK8	AlyWAK12	ArhWAK11	
4	BoWAK10	BnWAK12	AalWAK1	ArhWAK1	CsaWAK3	AtWAK3
5	BrWAK13	BnWAK11	BjuWAK9	BoWAK9		
6	ArhWAK5	ArhWAK9	BjuWAK10	BjuWAK19	BoWAK11	
7	BoWAK6	CsaWAK14	CsaWAK7	CsaWAK11	CsaWAK26	CsaWAK2
8	BnWAK22	BnWAK29	BnWAK32	AtWAK9	AlyWAK10	
9	ArhWAK7	AtWAK13	BrWAK1	AlyWAK8		
10	AalWAK3	AtWAK11	BrWAK4	ArhWAK4	BnWAK10	AtWAK7
11	AlyWAK6	ArhWAK10	AtWAK1	AlyWAK14		
12	BoWAK13	BnWAK30	BnWAK5	AlyWAK6	ArhWAK3	
13	BjuWAK16	BoWAK20	BnWAK3			
14	BjuWAK5	BjuWAK11	CsaWAK6	CsaWAK29	AalWAK6	
15	AtWAK4	BrWAK11	AlyWAK5	BoWAK3	BnWAK9	
16	AtWAK12	AlyWAK9	ArhWAK6	BjuWAK17		
17	BoWAK15	BnWAK2				
18	CsaWAK9	CsaWAK23	CsaWAK16	BnWAK7	BnWAK1	BnWA24 AalWAK4
19	BrWAK12	BjuWAK2	AlyWAK13	BrWAK9	BoWAK12	
20	BrWAK2	ArhWAK14				
21	CsaWAK12	CsaWAK27	CsaWAK5	AalWAK2	AtWAK10	BrWAK7
22	AlyWAK1	BoWAK2	BnWAK15	ArhWAK12	BoWAK4	
23	AtWAK10					
24	CsaWAK13	CsaWAK19	AtWAK6	AlyWAK7	ArhWAK16	
25	BjuWAK14	BoWAK1	CsaWAK21	BnWAK14	BrWAK10	BoWAK26
26	BnWAK25					
27	AtWAK8	AlyWAK4	ArhWAK2			
28	CsaWAK24	CsaWAK17				
29	BrWAK6	BnWAK4				
30	BrWAK3	BnWAK6				
31	AlyWAK2	ArhWAK13				
32	BjuWAK4	BnWAK27				
33	BoWAK19	BnWAK14				
34	BoWAK21	BnWAK23				
35	BoWAK23	BnWAK26				
36	BoWAK18	BnWAK20				
37	BoWAK17	BnWAK19				
38	BoWAK8	BnWAK35				

Table S3. Primers Used for qRT-PCR.

	F-Primers	R-Primers
ACT2	TCTCACACTGTGCCAAT	TCCACGTCACACTTCAT
BnWAK2	GGATAAGCAACGTCAGGGTT	TCCGAGTACATGCTCATCCT
BnWAK5	CTCTGCTTCCACCGCTAAAA	CACTACCCATCTCCTGCAAC
BnWAK11	GCCCCTTTTTCATCACAGAGA	CTTGTTCTTGTTGTCCCTCCC
BnWAK12	GCGACACTGTTTTGAGAAGC	TCCTCTCCAGCTCAATCCAA
BnWAK15	GTTGCTGTGGATGTTGCAG	GGCTCGGTACTTCTCGTCTA
BnWAK26	CTAACATCGAGCCGAGCATT	AGGCCACTCTACTGTTTTGG
BnWAK27	GTTTATCCCCAACGCAGGAG	CTGACACCTTGGCTCGATAC
BnWAK35	GCAGTCGCATGAGTACGTTA	GCGACACTGTTTTGAGAAGC

Table S4. Reaction Conditions for qRT-PCR.

Stage 1	Initial Denaturation	Rep: 1	95℃	30 sec
Stage 2	Cycling Reaction	Reps: 40	95℃<br>60℃	10sec<br>30sec
Stage 3	Melting Curve	Rep: 1	95℃<br>60℃<br>95℃	15sec<br>60sec<br>15sec

Table S5. Reaction Conditions for qRT-PCR.

Sample	*BnWAK11*	*BnWAK12*	*BnWAK15*	*BnWAK2*	*BnWAK26*	*BnWAK27*	*BnWAK35*	*BnWAK5*	*ACT2*
0-1	22.59223	21.93345	22.93245	24.03833	22.76782	27.38668	21.31435	18.81321	18.13777
0-2	21.84729	21.82725	23.4343	25.1701	22.28863	28.75868	24.24953	22.00081	18.38161
0-3	20.69122	21.40042	23.42816	22.60113	23.09925	21.80853	22.89246	19.27342	20.09675
0-4	26.36357	26.73704	27.43942	26.5423	28.21928	31.42168	25.87652	22.18376	19.26345
0-5	27.32036	24.3316	26.161	27.44865	26.45037	31.08668	23.52783	22.51321	18.8695
0-6	26.59196	24.45535	26.20942	27.23213	25.56872	31.24168	22.61888	24.77163	18.36
24-1	22.23299	25.15524	23.88251	24.0865	23.65051	27.78267	17.74112	21.22921	18.50721
24-2	23.60437	25.14873	25.64889	26.21787	24.77457	27.53237	21.33729	24.49988	19.90067
24-3	23.38976	24.16643	23.74751	24.56209	23.14257	26.39737	21.26974	21.46522	19.79588
24-4	23.60437	24.86528	24.88251	26.93871	19.72465	28.78267	22.40474	27.1065	20.93088
24-5	19.85265	21.07987	19.41614	21.93871	21.89273	22.17336	16.73121	18.79796	20.33873
24-6	19.85724	19.50843	19.00551	22.41504	21.01573	16.32722	19.04039	20.43963	18.4565
48-1	21.16863	18.93235	22.31446	21.48313	19.48358	23.65988	17.65366	19.41065	18.20891
48-2	21.23363	19.0413	21.07339	22.41601	19.52834	23.72488	17.59121	18.26987	18.07976
48-3	25.34062	22.52835	22.24564	21.87229	22.78352	25.65988	16.87643	20.76526	18.10206
48-4	25.90472	22.13904	22.3101	23.08755	23.46334	27.90049	19.84678	21.45915	18.97226
48-5	24.72153	23.78601	24.38003	23.52429	21.14316	26.65831	17.36905	20.82304	19.31913
48-6	16.64038	20.28501	21.53663	20.32686	20.59705	24.91494	18.15056	18.3836	20.96958
